# RAGE and ICAM-1 differentially control leukocyte recruitment during acute inflammation in a stimulus-dependent manner

**DOI:** 10.1186/1471-2172-12-56

**Published:** 2011-10-04

**Authors:** David Frommhold, Anna Kamphues, Susanne Dannenberg, Kirsten Buschmann, Victoria Zablotskaya, Raphaela Tschada, Baerbel Lange-Sperandio, Peter P Nawroth, Johannes Poeschl, Angelika Bierhaus, Markus Sperandio

**Affiliations:** 1 Department of Neonatology, University of Heidelberg, 69120 Heidelberg, Germany; 2 Department of Medicine I and Clinical Chemistry, University of Heidelberg, 69120 Heidelberg, Germany; 3 Dr. von Haunersches Kinderspital Ludwig-Maximilians-University, 81377 München, Germany; 4 Walter Brendel Center of Experimental Medicine, Ludwig-Maximilians-University, 81377 München, Germany

## Abstract

**Background:**

The receptor for advanced glycation endproducts, RAGE, is involved in the pathogenesis of many inflammatory conditions, which is mostly related to its strong activation of NF-κB but also due to its function as ligand for the β_2_-integrin Mac-1. To further dissect the stimulus-dependent role of RAGE on leukocyte recruitment during inflammation, we investigated β_2_-integrin-dependent leukocyte adhesion in *RAGE^-/- ^*and *Icam1^-/- ^*mice in different cremaster muscle models of inflammation using intravital microscopy.

**Results:**

We demonstrate that RAGE, but not ICAM-1 substantially contributes to N-formyl-methionyl-leucyl-phenylalanine (fMLP)-induced leukocyte adhesion in TNF-α-pretreated cremaster muscle venules in a Mac-1-dependent manner. In contrast, fMLP-stimulated leukocyte adhesion in unstimulated cremaster muscle venules is independent of RAGE, but dependent on ICAM-1 and its interaction with LFA-1. Furthermore, chemokine CXCL1-stimulated leukocyte adhesion in surgically prepared cremaster muscle venules was independent of RAGE but strongly dependent on ICAM-1 and LFA-1 suggesting a differential and stimulus-dependent regulation of leukocyte adhesion during inflammation in vivo.

**Conclusion:**

Our results demonstrate that RAGE and ICAM-1 differentially regulate leukocyte adhesion in vivo in a stimulus-dependent manner.

## Background

Leukocyte recruitment into inflamed tissue is considered a fundamental part of the inflammatory process and therefore plays a crucial role in immune defence. Leukocyte recruitment follows a well-defined cascade of events, beginning with the capture of free-flowing leukocytes to the vessel wall, followed by rolling, integrin-mediated firm adhesion to the endothelial layer, postarrest modifications and finally transmigration into tissue [1]. Firm adhesion of leukocytes to the endothelium crucially depends on the β_2_-integrins LFA-1 (CD11a/CD18, α_L_β_2_) and Mac-1 (CD11b/CD18, α_M_β_2_) which interact with different endothelial ligands such as ICAM-1 and RAGE, the receptor for advanced glycation end products [1-4]. Deficiency of the β_2_-integrin CD18, an essential part of both β_2_-integrins LFA-1 and Mac-1, leads to severe recurrent acute and chronic infections in mice and humans, which is caused by impaired leukocyte adhesion [5-7]. Interestingly, deficiency of either LFA-1 or Mac-1 in mice only mildly affects leukocyte recruitment in vivo indicating overlapping functions of LFA-1 and Mac-1 in mediating firm leukocyte adhesion [3,8]. Similarly, *Icam1 *knockout mice show only marginal inflammatory defects [9]. Recently, we demonstrated in a model of acute trauma-induced inflammation in the mouse, that the concomitant absence of ICAM-1 and RAGE leads to a dramatic decrease in leukocyte adhesion when compared to control mice or mice where only ICAM-1 or RAGE is absent [3]. These findings provide evidence that the integrin ligands ICAM-1 and RAGE exert overlapping functions [3]. At present it is unclear, if the adhesion molecules RAGE and ICAM-1 cooperate in a similar fashion in other models of inflammation. This prompted us to further dissect the role of ICAM-1 and RAGE for firm leukocyte adhesion under different inflammatory conditions. Using intravital microscopy, we observed leukocyte adhesion in cremaster muscle venules of *RAGE *and *Icam1 *knockout mice during trauma- or TNF-α-induced inflammation and additional local stimulation with the leukocyte chemoattractant peptide N-formyl-methionyl-leucyl-phenylalanine (fMLP) or systemic injection of the chemokine CXCL1. Our findings expand previous reports in as much as they show that besides its overlapping function, ICAM-1 and RAGE also exhibit distinct and stimulus-dependent functions in mediating leukocyte adhesion in vivo.

## Results

### Leukocyte adhesion in trauma-induced inflammation

**Figure 1:**
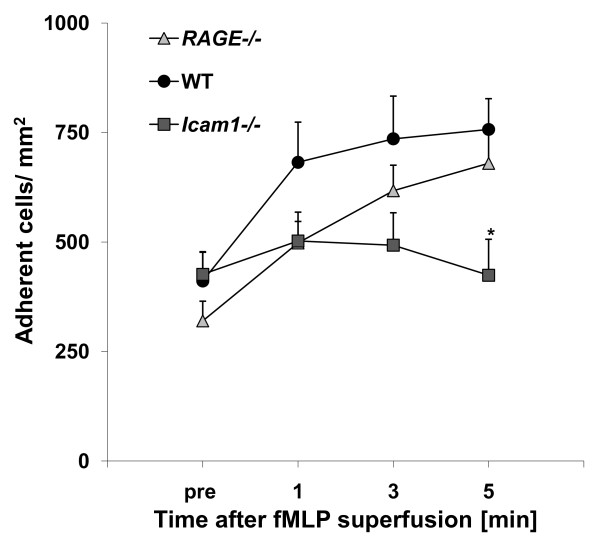
**Leukocyte adhesion in trauma-induced inflammation of cremaster muscle venules during additional fMLP superfusion**. Leukocytes adhesion (number of adherent cells/mm^2^) was observed in trauma-stimulated cremaster muscle venules of wild type (WT) control mice (13 venules in 10 mice), *RAGE*^-/- ^mice(10 venules in 6 mice) and *Icam1*^-/- ^mice (4 venules in 4 mice) before and during 5 minutes fMLP-superfusion (10 μM). All values are presented as mean ± SEM and significant differences (p < 0.05) vs. WT control mice are indicated by the asterisk.

During trauma-induced inflammation, which is induced by surgical preparation, firm leukocyte arrest is mostly mediated via the β_2_-integrins LFA-1 and Mac-1 interacting with ICAM-1 and RAGE, respectively [3]. Here, we observed leukocyte adhesion in postcapillary venules of the surgically prepared cremaster muscle of wild type, *RAGE^-/- ^*and *Icam1^-/- ^*mice before and during fMLP superfusion. Microvascular and hemodynamic parameters were similar between genotypes and treatment groups and there were no significant changes of systemic leukocyte counts during fMLP superfusion (Table 1). In line with an earlier report [3], leukocyte adhesion in trauma-stimulated cremaster muscle venules was similar in wild type mice, *Icam1^-/- ^*mice and *RAGE^-/- ^*mice prior to fMLP superfusion (Figure 1). To explore the contribution of RAGE and ICAM-1 in mediating firm leukocyte adhesion in response to fMLP, we observed leukocyte adhesion in postcapillary venules of the cremaster muscle during 5 min fMLP superfusion. fMLP produced a strong increase in leukocyte adhesion in both *RAGE^-/- ^*mice and wild type mice. In contrast, fMLP-stimulated leukocyte adhesion was significantly attenuated in *Icam1^-/- ^*mice suggesting that fMLP-induced leukocyte adhesion in surgically prepared cremaster muscle venules is dependent on ICAM-1, but independent of RAGE (Figure 1).

**Table 1:** Hemodynamic and microvascular parameters in cremaster muscle venules during trauma induced inflammation and additional fMLP stimulation

	Mice	Venules	Diameter	Centerline Velocity	Wall shear rate	Systemic leukocyte counts
	(N)	(n)	(μm)	(μm/s)	(s-1)	(cells/μl)
						
Wild type	10	13	33 ± 1	2700 ± 200	2000 ± 100	6600 ± 600
*RAGE^-/-^*	6	10	33 ± 2	2700 ± 200	2100 ± 100	6800 ± 900
*Icam1^-/-^*	4	4	30 ± 2	2400 ± 300	2000 ± 300	6400 ± 300

Wild type+ anti-Mac-1	6	19	30 ± 1	2500 ± 100	2100 ± 100	4800 ± 200
*RAGE-^/-^*+ anti-Mac-1	4	14	29 ± 2	2500 ± 100	2100 ± 150	6700 ± 600
Wild type+ isotype AB	3	9	33 ± 2	2300 ± 100	1800 ± 100	6300 ± 700

Wild type+ anti-LFA-1	5	16	27 ± 1	2400 ± 100	2200 ± 100	6900 ± 700
*RAGE-/-*+ anti-LFA-1	6	20	30 ± 1	2400 ± 100	2200 ± 100	7900 ± 700
Wild type+ isotype AB	3	9	29 ± 2	2400 ± 100	2200 ± 200	5300 ± 400
			n.s.	n.s.	n.s.	n.s.

Vessel diameter, centerline velocity, wall shear rate and systemic leukocyte counts are presented as mean ± SEM of the investigated venules during trauma-induced inflammation with additional local fMLP superfusion. N.s. = non significant.

### Role of Mac-1 and LFA-1 for leukocyte adhesion in fMLP-stimulated trauma-induced inflammation

**Figure 2:**
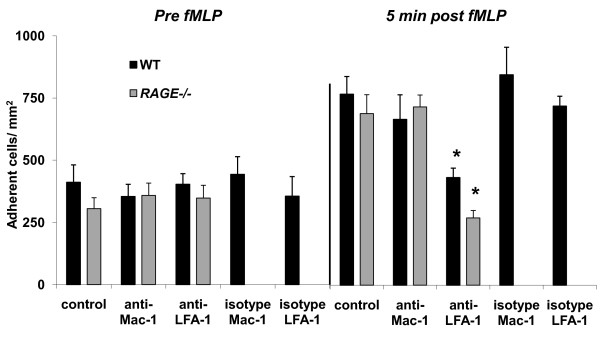
**Role of Mac-1 and LFA-1 for leukocyte adhesion in surgically prepared cremaster muscle venules before and after additional fMLP superfusion**. Leukocytes adhesion (number of adherent cells/mm^2^) is shown before and 5 minutes after fMLP-superfusion (10 μM) in surgically prepared cremaster muscle venules of wild type mice (13 venules in 10 mice) and *RAGE*^-/- ^mice (10 venules in 6 mice) without antibody treatment and after they were systemically treated with blocking antibodies against murine Mac-1 (Tib128; 19 venules in 6 wild type mice and 14 venules in 4 *RAGE*^-/- ^mice) or LFA-1 (Tib217, 16 venules in 5 wild type mice and 20 venules in 6 *RAGE*^-/- ^mice), or their respective isotype control antibodies (IgG2b for Mac-1; 11 venules in 4 wild type mice and IgG2a for LFA-1; 12 venules in 4 wild type mice) immediately before starting fMLP superfusion. All values are presented as mean ± SEM and significant differences (p < 0.05) vs. control mice are indicated by the asterisk.

To further dissect the role of β_2_-integrins for fMLP-stimulated leukocyte adhesion, we investigated fMLP-induced leukocyte adhesion in exteriorized cremaster muscle venules of wild type and *RAGE*^-/- ^mice pretreated with blocking mAbs against the β_2_-integrins Mac-1 or LFA-1 using anti Mac-1 mAb Tib128 (clone M1/70) and anti LFA-1 mAb Tib217 (clone M17/4), respectively. To rule out unspecific effects of the antibodies we injected wild type mice with respective isotype control antibodies. It is important to mention, that we injected all antibodies immediately before fMLP superfusion to identify the role of β_2_-integrins for fMLP induced leukocyte adhesion only. Hemodynamic and microvascular parameters did not vary significantly between the different groups (Table 1). Before fMLP superfusion leukocyte adhesion was similar in trauma-stimulated cremaster muscle venules of wild type mice and *RAGE^-/- ^*mice in response to different antibody treatment (Figure 2). This is an expected finding due to the fact that antibodies were injected after induction of trauma. The fMLP-induced increase of leukocyte adhesion (five minutes after fMLP) in wild type or *RAGE*^-/- ^mice was not affected by pretreatment with Mac-1-blocking mAbTib128. However, fMLP-induced leukocyte adhesion was almost absent in wild type mice or *RAGE*^-/- ^mice pretreated with LFA-1-blocking mAb Tib217 (Figure 2) suggesting that fMLP-stimulated adhesion is dependent on LFA-1 and ICAM-1 but independent of RAGE and Mac-1.

### Leukocyte adhesion in TNF-α-induced inflammation

**Figure 3:**
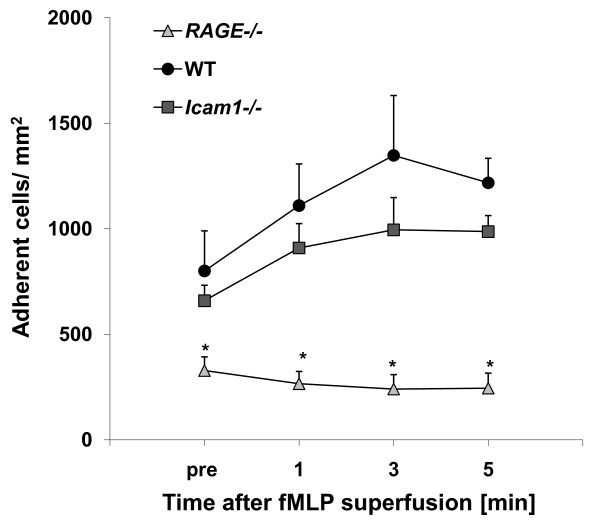
**Leukocyte adhesion in TNF-α-stimulated cremaster muscle venules during additional fMLP superfusion**. Leukocytes adhesion (number of adherent cells/mm^2^) was observed in 3 h TNF-α-stimulated cremaster muscle venules of wild type (WT) control mice (6 venules in 6 mice), *RAGE*^-/- ^mice (5 venules in 5 mice) and *Icam1*^-/- ^mice (4 venules in 4 mice) before and during 5 minutes fMLP-superfusion (10 μM). All values are presented as mean ± SEM and significant differences (p < 0.05) vs. WT control mice are indicated by the asterisk.

Leukocyte adhesion in TNF-α-stimulated cremaster muscle venules was observed by intravital microscopy in six wild type mice, five *RAGE^-/- ^*mice, and four *Icam1^-/- ^*mice. Hemodynamic and microvascular parameters did not vary significantly between the different groups (Table 2). As described earlier, leukocyte adhesion was significantly reduced in *RAGE^-/- ^*mice compared to wild type mice or *Icam1^-/- ^*mice prior to fMLP superfusion (Figure 3). Since fMLP is known to trigger leukocyte adhesion in TNF-α-induced inflammation independent of ICAM-1 [10], we wanted to test if fMLP-induced leukocyte adhesion in TNF-α-treated cremaster muscle venules is dependent on RAGE. Similar to the report by Foy and Ley [10], we observed an increase in leukocyte adhesion during five minutes of fMLP-superfusion in TNF-α-treated cremaster muscle venules of *Icam1*^-/- ^mice and wild type mice. However, there was no fMLP-induced increase in leukocyte adhesion in *RAGE^-/- ^*mice (Figure 3), suggesting that leukocyte adhesion is independent of ICAM-1, but dependent on RAGE in this setting.

**Table 2:** Hemodynamic and microvascular parameters in cremaster muscle venules during TNF-α-induced inflammation

	Mice	Venules	Diameter	Centerline Velocity	Wall shear rate	Systemic leukocyte counts
	(N)	(n)	(μm)	(μm/s)	(s^-1^)	(cells/μl)
						
Wild type	6	6	30 ± 3	2200 ± 200	1800 ± 100	6300 ± 1000
*RAGE^-/-^*	5	5	32 ± 2	2800 ± 200	2200 ± 200	4200 ± 700
*Icam1^-/-^*	4	4	30 ± 2	2400 ± 300	2000 ± 300	6400 ± 300

Wild type + anti-Mac-1	4	20	29 ± 2	2200 ± 100	2000 ± 100	4200 ± 200
*Icam1^-/- ^*+ anti-Mac-1	3	13	30 ± 2	2500 ± 100	2000 ± 100	4800 ± 400
Wild type + isotype	3	10	30 ± 2	2500 ± 100	2100 ± 100	4000 ± 500

Wild type + anti-LFA-1	4	13	28 ± 2	2400 ± 100	2100 ± 100	4900 ± 300
*Icam1^-/- ^*+ anti-LFA-1	3	14	27 ± 3	2200 ± 200	2400 ± 400	5000 ± 300
Wild type + isotype	3	9	31 ± 2	2500 ± 100	2100 ± 200	4600 ± 300
			n.s.	n.s.	n.s.	n.s.

Vessel diameter, centerline velocity, wall shear rate and systemic leukocyte counts are presented as mean ± SEM of the investigated venules during TNF-α-induced inflammation with additional local fMLP superfusion and after anti-Mac-1 and anti-LFA-1 antibody treatment. n.s. = non significant.

### Role of Mac-1 and LFA-1 for fMLP-induced leukocyte adhesion in TNF-α-stimulated-cremaster muscle venules

**Figure 4:**
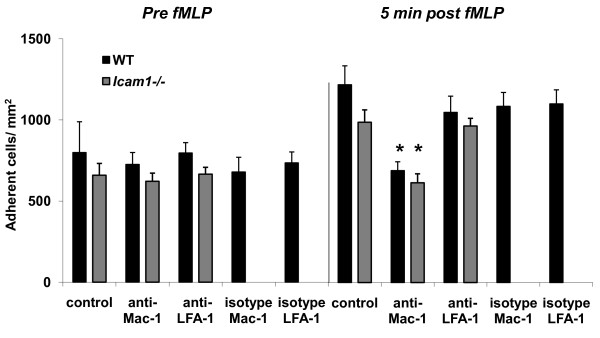
**Role of Mac-1 and LFA-1 for leukocyte adhesion in TNF-α-stimulated cremaster muscle venules before and after additional fMLP superfusion**. Leukocytes adhesion (number of adherent cells/mm^2^) is shown before and 5 minutes after fMLP-superfusion (10 μM) in 3 h TNF-α-stimulated cremaster muscle venules of wild type control mice and *Icam1*^-/- ^mice, which are systemically treated with blocking antibodies against murine Mac-1 (Tib128; 20 venules in 4 wild type mice and 13 venules in 3 *Icam1*^-/- ^mice), LFA-1 (Tib217, 13 venules in 4 wild type mice and 14 venules in 3 *Icam1*^-/- ^mice) or their respective isotype control antibodies (IgG2b for Mac-1; 10 venules in 3 wild type mice and IgG2a for LFA-1; 8 venules in 3 wild type mice) immediately before starting fMLP superfusion or without antibody pretreatment (6 venules in 6 wild type mice and 4 venules in 4 *Icam1*^-/- ^mice). All values are presented as mean ± SEM and significant differences (p < 0.05) vs. WT control mice and *Icam1*^-/- ^control mice are indicated by the asterisk.

To further explore the adhesion molecules involved in fMLP-induced leukocyte adhesion of TNF-α-stimulated cremaster muscle venules, we investigated fMLP-induced leukocyte adhesion in TNF-α-treated cremaster muscle venules using blocking mAbs against Mac-1 and LFA-1. To rule out unspecific effects of the antibodies, we injected wild type mice with respective isotype control antibodies. In parallel to the trauma model, we injected all antibodies immediately before fMLP superfusion to identify the role of β_2_-integrins for fMLP-induced leukocyte adhesion only. Hemodynamic and microvascular parameters were similar between the different groups (Table 2). As expected in this setting, before fMLP superfusion leukocyte adhesion was similar in TNF-α-stimulated cremaster muscle venules of wild type mice and *Icam1^-/- ^*mice in the different treatment groups (Figure 4). Five minutes after fMLP superfusion the fMLP-induced increase of leukocyte adhesion was absent in wild type treated with the Mac-1-blocking mAb Tib128 shortly before fMLP superfusion (Figure 4). In contrast, in wild type mice treatment with LFA-1-blocking antibody Tib217 did not alter fMLP-induced leukocyte adhesion compared to wild type mice without antibody treatment, suggesting that fMLP-induced leukocyte adhesion in this setting is mostly dependent on Mac-1 but not LFA-1. Accordingly, treatment of *Icam1^-/- ^*mice with Mac-1-blocking mAb Tib128 abolished fMLP-triggered leukocyte adhesion, while the absence of ICAM-1 (with or without blockade of LFA-1) did not influence the fMLP-induced increase in leukocyte adhesion (Figure 4). These findings suggest that fMLP-induced leukocyte adhesion in TNF-α-treated cremaster muscle venules is dependent on RAGE and Mac-1, whereas ICAM-1 and LFA-1 are not required.

### Leukocyte adhesion following systemic stimulation by chemokine CXCL1 in trauma-induced inflammation

**Figure 5:**
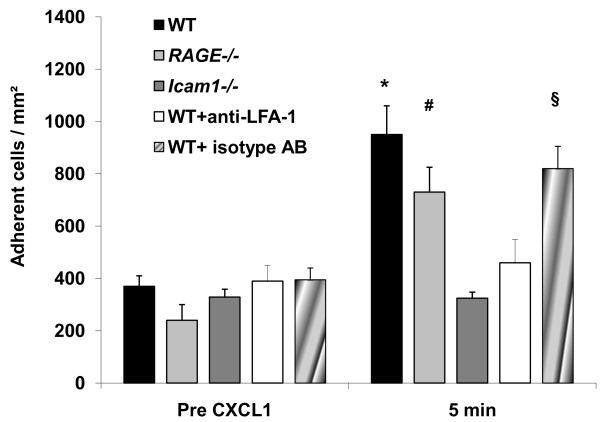
**Leukocyte adhesion in CXCL1-stimulated cremaster muscle venules**. The number of adherent cells (mean ± SEM) was assessed in exteriorized cremaster muscle venules of *RAGE*^-/- ^mice (10 venules in 6 mice), *Icam1^-/- ^*mice (11 venules in 5 mice), untreated (12 venules in 7 mice), anti-LFA-1 antibody (10 venules in 4 mice) or isotype antibody (9 venules in 3 mice) treated wild type mice before and five minutes after systemic injection of CXCL1 (keratinocyte-derived chemokine, KC; 600 ng/mouse). Significant differences (p < 0.05) of the number of adherent leukocytes before CXCL1 vs. after injection of CXCL1 are indicated by *, # and § for wild type mice, *RAGE*^-/- ^mice and isotype antibody treated wild type mice, respectively.

To investigate a potential role of RAGE, ICAM-1 or LFA-1 in chemokine-induced firm leukocyte arrest in vivo, we systemically injected the CXCR2 chemokine CXCL1 (KC) into *RAGE^-/- ^*and *Icam1^-/- ^*mice, and into wild type mice with and without anti-LFA-1-blocking or isotype control antibody pretreatment. Systemic injection of CXCL1 has been shown to induce firm leukocyte adhesion in exteriorized cremaster muscle venules of wild type mice [11,12]. Hemodynamic and microvascular parameters were similar between the different groups (Table 3). Following systemic injection of CXCL1, we observed a significant increase in the number of adherent cells in wild type mice (Figure 5, p < 0.05). Similarly, in *RAGE*-deficient mice the number of adherent cells significantly increased five minutes after CXCL1 injection (Figure 5, p < 0.05), suggesting that CXCL-1-triggered activation of β_2_-integrins leading to firm leukocyte arrest does not require RAGE. In contrast, in *Icam1^-/- ^*mice, systemic injection of CXCL1 did not lead to an increase in leukocyte adhesion (Figure 5) indicating that CXCL1-triggered leukocyte arrest is strongly dependent on ICAM-1. Furthermore, pretreatment of wild type mice with the anti-LFA-1-blocking antibody Tib 217, but not its isotype control antibody, inhibited CXCL1-induced leukocyte arrest, suggesting that LFA-1 and ICAM-1 play a predominant role in mediating CXCL1-triggered leukocyte arrest during trauma-induced inflammation in vivo.

**Table 3:** Hemodynamic and microvascular parameters in cremaster muscle venules during trauma induced inflammation with additional CXCL1 stimulation

	Mice	Venules	Diameter	Centerline Velocity	Wall shear rate	Systemic leukocyte counts
	(N)	(n)	(μm)	(μm/s)	(s-1)	(cells/μl)
						
Wild type	7	12	32 ± 3	2200 ± 300	1600 ± 200	6300 ± 500
*RAGE*^-/-^	6	10	32 ± 3	2000 ± 200	1800 ± 200	7400 ± 800
*Icam-1^-/-^*	4	11	28 ± 1	2400 ± 100	2100 ± 100	8000 ± 800
Wild type+ anti-LFA-1	4	10	28 ± 2	2400 ± 100	2200 ± 100	7000 ± 500
Wild type+ Isotype AB	3	9	29 ± 2	2500 ± 200	2200 ± 100	7500 ± 600
			n.s.	n.s.	n.s.	n.s.

Vessel diameter, centerline velocity, wall shear rate and systemic leukocyte counts are presented as mean ± SEM of the investigated venules during trauma-induced inflammation and systemic injection of the chemokine CXCL1 (KC). N.s. = non significant.

### Stimulus-dependent expression of adhesion molecules in cremaster muscle venules

**Figure 6:**
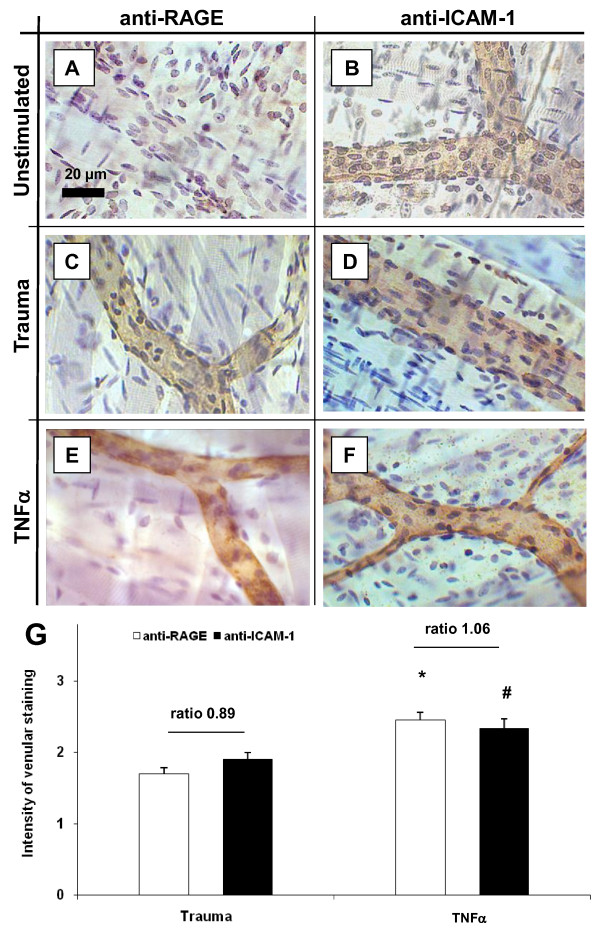
**Stimulus-dependent expression of RAGE and ICAM-1 in cremaster muscle venules**. Immunostaining was conducted to assess endothelial expression of RAGE (A, C, E) and ICAM-1 (B, D, F) in postcapillary venules of cremaster muscles obtained directly postmortem (Unstimulated, upper panel), after exteriorization and 20 min superfusion (Trauma, middle panel), or after 3 h of TNF-α-stimulation and following exteriorization (TNF-α, lower panel; at least 3 mice/group). Application of primary antibody was performed i.v. before harvesting the cremaster muscle in order to stain RAGE and ICAM-1 on the endothelial surface. Biotinylated secondary antibody, peroxidase-conjugated streptavidin and diaminobenzidine (DAB) were used to detect endothelial expression of ICAM-1 and RAGE as brown signal. Counter-staining was performed by Mayer's hemalaun. Reference bar for the representative images is shown in A and represents 20 μm. Intensity of venular anti-RAGE and anti-ICAM-1 immunostaining during trauma- and TNF-α-induced inflammation were analyzed semiquantitatively and presented as mean ± SEM (G; 0 = no, 1 = weak, 2 = medium, 3 = strong signal). Significant differences (p < 0.05) of TNF-α-induced RAGE or ICAM-1 expression vs. trauma are indicated by the asterisk or the pound key, respectively. Relative ratios of expression of RAGE to ICAM-1 are quantified for the trauma and TNF-α model as illustrated.

Next, we addressed the question whether the stimulus-dependent role of ICAM-1-LFA vs. RAGE-Mac-1 for leukocyte adhesion is due to altered endothelial expression of adhesion molecules. Immunohistochemistry was used to assess endothelial expression of ICAM-1 and RAGE in postcapillary venules of cremaster muscles obtained from wild type mice either directly postmortem (unstimulated), after exteriorization and superfusion (trauma model) or after 3 h of TNF-α-stimulation followed by surgical preparation (TNF-α model). While we found no RAGE expression on the endothelium of unstimulated cremaster muscle venules of wild type mice (Figure 6A), after trauma and even more pronounced during TNF-α-induced inflammation, RAGE expression could be clearly detected (Figure 6C and 6E, respectively). As illustrated in Figure 6B, D, F, we found endothelial surface expression of ICAM-1 in unstimulated, trauma- and TNF-α stimulated cremaster muscle venules.

Beside representative micrographs, the immunostaining were scored semi-quantitatively to compare endothelial expression of RAGE to ICAM-1 between the inflammatory models (Figure 6G). During trauma-induced inflammation we found a moderate expression of RAGE, which was slightly below that of ICAM-1 resulting in a RAGE/ICAM-1 expression ratio of 0.89. TNF-α-stimulation produced a significant upregulation of both RAGE and ICAM-1 compared to trauma-induced inflammation. However, the increase of RAGE expression was stronger than for ICAM-1, which is reflected by a RAGE/ICAM-1 expression ratio of 1.06 indicating that expression of RAGE is more strongly inducible by inflammatory stimulation than expression of ICAM-1.

### Stimulus-dependent expression of LFA-1 and Mac-1

**Figure 7:**
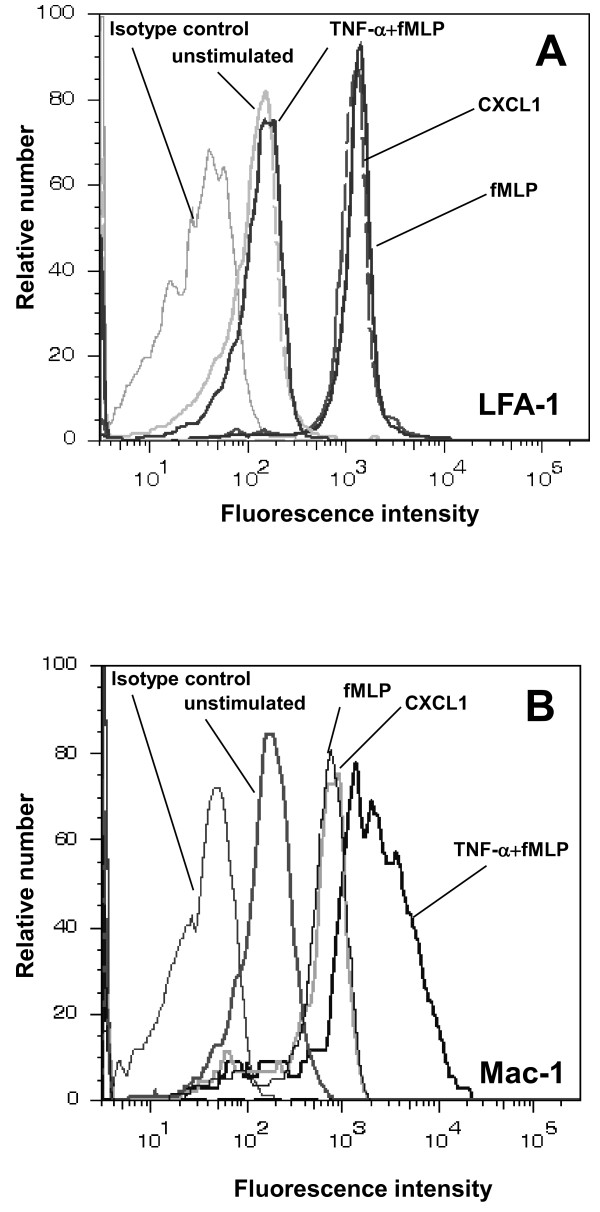
**Stimulus-dependent expression of LFA-1 and Mac-1 on neutrophils**. Surface expression of LFA-1 (A) and Mac-1 (B) on bone marrow-derived neutrophils (n = 3 mice) after stimulation with CXCL1 (KC; 120 ng per 10^6 ^leukocytes/ml, 15 min), fMLP (10 μM, 10^6 ^leukocytes/ml, 15 min), or TNF-α+ fMLP (25 ng TNF-α for 3 h followed by 10 μM fMLP for 15 min in 10^6 ^leukocytes/ml) was compared to unstimulated controls (B). Representative histograms are shown from 3 separate experiments.

To further explore underlying mechanisms, we investigated Mac-1 and LFA-1 expression on isolated wild type neutrophils pretreated with TNF-α, fMLP, or CXCL1 using flow cytometry. LFA-1 expression on wild type neutrophils was strongly upregulated after fMLP and CXCL-1 treatment compared to baseline expression (Figure 7A). However, neutrophils pretreated with TNF-α followed by fMLP treatment only showed a mild increase in LFA-1 expression (Figure 7A). In contrast, expression of Mac-1 was only mildly increased in response to fMLP or CXCL-1, but increased strongly upon stimulation with TNF-α and fMLP when compared to unstimulated neutrophils (Figure 7B). These results demonstrate that LFA-1 and Mac-1 expression are differentially affected by the various stimuli which is in line with the in vivo results shown in Figure 2 and 4.

## Discussion

The receptor of advanced glycation endproducts RAGE belongs to the pattern recognition receptors and exerts its central function during acute and chronic inflammations not only as a strong activator of the proinflammatory transcription factor NF-κB but also as endothelial-expressed ligand for the β_2_-integrin Mac-1 [3,4,13]. The biological relevance of RAGE as a ligand for Mac-1 has been recently demonstrated in *RAGE*^-/- ^× *Icam1*^-/- ^mice where leukocyte adhesion was almost completely absent in an acute surgically-induced model of inflammation in the cremaster muscle in vivo [3] suggesting that RAGE and ICAM-1 cooperate closely in mediating firm leukocyte adhesion, with RAGE interacting with Mac-1 and ICAM-1 binding to LFA-1. Interestingly, the same study revealed that under circumstances where inflammation was induced by local treatment with TNF-α plus the surgical trauma, leukocyte adhesion was only mildly reduced in the absence of RAGE and ICAM-1 suggesting that alternative, yet unknown integrin ligands exist and that the close cooperation of RAGE and ICAM-1 as β_2_-integrin ligands is stimulus-dependent (Table 4) [3]. To further investigate the role of RAGE as Mac-1 ligand during leukocyte recruitment, we studied here leukocyte adhesion in cremaster muscles of *RAGE^-/-^, Icam1^-/- ^*and wild type mice using different inflammatory models in the cremaster muscle. We show that additional fMLP stimulation during trauma-induced inflammation leads to increased leukocyte adhesion which is independent of RAGE but dependent on ICAM-1, while fMLP superfusion in TNF-α pretreated cremaster muscle venules induces RAGE-dependent adhesion which was independent of ICAM-1 (Table 4). Additional studies using mAbs against Mac-1 and LFA-1 confirmed that RAGE interacts with Mac-1 and ICAM-1 with LFA-1, respectively. These findings imply that the same pro-inflammatory agent (i.e. fMLP) can lead to different biological effects. This is also supported by an earlier report from Foy and Ley [10] showing that fMLP-induced leukocyte adhesion in vivo is only dependent on ICAM-1 in exteriorized cremaster muscles without any other stimulus while additional prestimulation with TNF-α leads to an ICAM-1-independent increase in leukocyte adhesion following fMLP superfusion [10]. The molecular mechanisms responsible for the differential and stimulus-dependent regulation of β_2_-integrin-mediated leukocyte adhesion are currently unclear.

**Table 4:** Stimulus-dependent regulation of RAGE and ICAM-1-mediated firm leukocyte adhesion in inflamed postcapillary venules of the cremaster muscle

	*Trauma-induced inflammation*			*TNF-α-induced inflammation*	
Stimulation:	*None*	*fMLP*	*KC*	*None*	*fMLP*
**ICAM-1 - LFA-1**	**+**^b^	**+**^a, c^	**+**^a^	**-**^b^	**-^a, c^**
**RAGE - Mac-1**	**+**^b^	**-**^a^	**-**^a^	**+**^b^	**+**^a^
**Additional yet unknown integrin ligands**	**-**^b^	**-**^a^	**-**^a^	**+**^b^	**-**^a^

**^a ^**this study, **^b ^**reference [3], **^c ^**in part reference [10]

On the endothelial side, our immunohistochemical findings showed a stimulus-dependent expression of ICAM-1 and RAGE. During trauma-induced inflammation, endothelial RAGE expression was less than ICAM-1 expression, while after TNF-α treatment RAGE expression on the inflamed endothelium was slightly higher than ICAM-1 expression. However, these subtle differences in expression might not explain, why fMLP-induced leukocyte adhesion relies on LFA-1/ICAM-1 during trauma-induced inflammation and on Mac-1/RAGE during TNF-α-stimulated inflammation.

In contrast, our flow cytometric analysis on the expression of Mac-1 and LFA-1 showed that LFA-1 expression on wild type neutrophils was profoundly increased after CXCL1 or fMLP treatment, while pretreatment with TNF-α followed by fMLP did not alter LFA-1 expression. Inversely, Mac-1 upregulation was most prominent on TNF-α plus fMLP treated neutrophils in comparison to CXCL1 or fMLP treatment alone. These findings are in line with our in vivo findings and also to previous observations showing that TNF-α induces Mac-1, but not LFA-1 expression on neutrophils [14,15].

Besides these more quantitative changes in the expression of Mac-1 and LFA-1 on PMN, additional mechanisms could lie in qualitative changes, i.e. in a stimulus-dependent activation of β_2_-integrins differing between Mac-1 and LFA-1 [16]. Our group recently showed that ICAM-1 binding to LFA-1 (but not to Mac-1) mediates firm leukocyte adhesion in inflamed cremaster muscle venules, whereas leukocyte crawling in the same setting is dependent on interactions between ICAM-1 and Mac-1 [3]. This suggests that during firm leukocyte arrest, different activation signals are necessary to enable interactions between LFA-1 and ICAM-1 or Mac-1 and ICAM-1. Indeed, different mechanisms of Mac-1 and LFA-1 activation are reported. Fagerholm and colleagues found that ICAM-1 binding to Mac-1 in neutrophils is regulated by α-chain phosphorylation of Mac-1 [17,18]. As Chatila et al. demonstrated in an earlier study that fMLP treatment of unstimulated PMN did not affect α-chain phosphorylation of Mac-1 [19], this might explain why Mac-1 does not significantly contribute to fMLP-triggered adhesion during trauma-induced inflammation. Noteworthy, LFA-1 and Mac-1 also differ in their binding site on ICAM-1. While LFA-1 uses the D3 domain on ICAM-1 for binding, Mac-1 recognizes the D5 domain on ICAM-1 [20,21].

Finally, posttranslational glycosylation has been reported as an important regulatory mechanism for leukocyte arrest and integrin-dependent adhesion [22]. For the β_2-_integrin Mac-1, Feng et al. demonstrated recently that activation of endogenous PMN sialidase leads to desialylation of Mac-1 with a subsequent increase in leukocyte adhesion [23]. The authors explained their findings by a better exposure of the activation epitope on Mac-1 upon desialylation of crucial sialic acid residues on Mac-1.

## Conclusions

Our study provides evidence that RAGE is a relevant, but context-dependent Mac-1 ligand during inflammation in vivo. In addition, the presented findings expand our view of the complex and stimulus-dependent regulation of leukocyte adhesion during inflammation (depicted in Table 4), which might lead to new RAGE-based strategies to specifically interfere with the recruitment of immune cells during specific acute and chronic inflammatory conditions.

## Methods

### Animals

*RAGE^-/- ^*mice and *Icam1^-/- ^*mice were generated as described earlier and backcrossed for at least seven generations into the C57BL/6 background [24,25]. All mice were maintained as breeding colonies at the Central Animal Facility of the University of Heidelberg, Germany. The animal experiments were approved by the Animal Care and Use Committee of the Regierungspräsidium Karlsruhe, Germany (AZ 35-9185.81/G-67/03 and AZ 35-9185.81/G-08/08).

### Antibodies and cytokines

In some intravital microscopic experiments, fMLP (10 μM; Sigma, Seisenhofen, Germany) was added to the superfusion buffer to induce additional leukocyte adhesion as described [26]. In certain experiments, recombinant murine TNF-α (R&D) was injected intrascrotally at a dose of 500 ng per mouse 3 hours before intravital microscopy. In some experiments, recombinant murine CXCR2 chemokine CXCL1 (keratinocyte-derived chemokine KC; Peprotech, London, UK) was injected systemically at a dose of 600 ng/mouse. Blocking antibodies against murine Mac-1 (Tib128, clone M1/70, rat IgG2b) and murine LFA-1 (Tib217, clone M17/4, rat IgG2a) were obtained from American Type Culture Collection (ATCC, Manasses, USA) and systemically administered at 100 μg/mouse immediately before starting fMLP superfusion.

### Intravital microscopy

Mice were prepared for intravital microscopy as reported recently [12]. Briefly, mice were anesthetized with intraperitoneal (i.p.) injection of ketamine (125 mg/kg body weight, Ketanest^®^, Pfizer GmBH, Karlsruhe, Germany) and xylazine (12.5 mg/kg body weight; Rompun^®^, Bayer, Leverkusen, Germany) and placed on a heating pad to maintain body temperature at 37°C. Intravital microscopy was conducted on an upright microscope (Leica; Wetzlar, Germany) with a saline immersion objective (SW40/0.75 numerical aperture, Zeiss, Jena, Germany). To ease breathing mice were intubated using PE 90 tubing (Becton Dickinson and Company, Sparks, MD, USA). The left carotid artery was cannulated with PE 10 tubing (Becton Dickinson and Company, Sparks, MD, USA) for blood sampling and systemic mAb administration.

### Cremaster muscle preparation

The surgical preparation of the cremaster muscle for intravital microscopy was performed as previously described [27]. Briefly, the scrotum was opened and the cremaster muscle was mobilized. After longitudinal incision and spreading of the muscle over a cover glass, the epididymis and testis were moved and pinned to the side giving full microscopic access to the cremaster muscle microcirculation. Cremaster muscle venules were recorded via CCD camera (CF8/1; Kappa, Gleichen, Germany) on a Panasonic S-VHS recorder. The cremaster muscle was superfused with thermocontrolled (35°C) bicarbonate-buffered saline. For local treatment with fMLP, 10 μM fMLP was added to the superfusion buffer and administrated over 15 min to the cremaster muscle tissue similar to a previously described protocol [26]. Postcapillary venules under observation were recorded before and during fMLP administration and ranged from 20-40 μm in diameter. Systemic blood samples (10 μl) were taken and assessed for white blood cell count before and after experiment. Blood samples were diluted 1:10 with Türck's solution (Merck, Darmstadt, Germany) and leukocytes concentration was expressed as number of leukocytes per microliter of whole blood using hematocytometer. Microvascular parameters (venular diameter, venular vessel segment length) were measured using an image processing system [28,29]. Venular centerline red blood cell velocity was measured during the experiment via a dual photodiode and a digital on-line cross-correlation program (Circusoft Instrumentation, Hockessin, USA). An empirical factor of 0.625 was used to convert centerline velocities to mean blood flow velocities [30]. Wall shear rates (γ_w_) were estimated as 4.9 (8vb/d), where vb is mean blood flow velocity and d the diameter of the vessel [31,32].

### Immunohistochemistry

To investigate the endothelial expression of RAGE and ICAM-1 on unstimulated cremaster muscle venules, during trauma- or TNF-α-induced inflammation, we performed immunohistochemical analysis of whole mount cremaster muscles as described [3]. Briefly, primary antibodies against RAGE (polyclonal rat anti mouse; 30 μg/mouse; Santa Cruz, Heidelberg, Germany) or ICAM-1 (YN-1, monoclonal rat anti mouse; 30 μg/mouse; ATCC) were systemically injected and incubated for 10 min ensuring staining of RAGE and ICAM-1 on the endothelial surface. Excess antibody was washed out from the circulation with normal saline solution either before scarifying the mouse (unstimulated) or after 20 min superfusion of the cremaster muscle (trauma- and TNF-α-stimulation). Cremaster muscle whole mounts were surgically prepared as reported previously [3] and transferred onto adhesive slides (Superfrost; Menzel, Germany). Staining of tissue samples for endothelial ICAM-1 and RAGE expression was performed using diaminobenzidine (DAB kit; Vector Lab, Burlingame, USA). Analysis of stained slides was conducted semiquantitatively in a blinded manner (0 = no, 1 = weak, 2 = medium, 3 = strong signal) on a Leica DMRB upright microscope and a ×25/0,75 NA oil immersion objective (both Leica, Wetzlar, Germany). Photographs of the samples were taken using a color CCD camera (KAPPA).

### Flow cytometry

The expression of Mac-1 and LFA-1 on bone marrow-derived neutrophils was assessed by flow cytometry as described previously [3]. After red blood cell lysis, 10^6 ^leukocytes/ml were stimulated for 15 min with 120 ng CXCL1 (KC) or 10 μM fMLP alone, or with 25 ng TNF-α over 3 h followed by 10 μM fMLP for 15 min (all at 37°C). Next, cells were incubated in the dark with FITC-conjugated anti- Mac-1 mAb M1/70 (1 μg/10^5 ^cells, rat IgG2b; eBioscience, San Diego, USA), FITC-conjugated LFA-1 mAb M17/4 (1 μg/10^5 ^cells, rat IgG2a; eBioscience, San Diego, USA) or respective FITC-conjugated isotype control antibodies (1 μg/10^5 ^cells, rat IgG2b or rat IgG2a; eBioscience, San Diego, USA) to detect anti-Mac-1 and LFA-1 signals, respectively. Mac-1 and LFA-1 expression was assessed on 10.000 cells/mouse within the neutrophil cluster defined by forward-side scatter analysis using LSRII with DIVA software package (Becton Dickinson, San Jose, USA). Expression of Mac-1 and LFA-1 upon stimulation with fMLP, CXCL1, or TNF-α followed by fMLP was compared to unstimulated cells and their respective isotype controls.

### Statistics

Sigma Stat 3.5 (Systat Software, Erkrath, Germany) was used for statistical analysis. Leukocyte counts, vessel diameters, centerline velocity, leukocyte adhesion and wall shear rates were compared with one-way ANOVA followed by a multiple pairwise comparison test (Dunn's test) or by Wilcoxon rank-sum test, as appropriate. Statistical significance was set at p < 0.05.

## Abbreviations

fMLP: N-formyl-methionyl-leucyl-phenylalanine; ICAM-1: intercellular adhesion molecule-1; KC: keratinocyte-derived chemokine (CXCL1); LFA-1: leukocyte functional antigen-1; Mac-1: macrophage antigen complex 1; NF-kB: Nuclear Factor kB; RAGE: receptor for advanced glycation endproducts; TNF-α: tumor necrosis factor-α.

## Competing interests

The authors declare that they have no competing interests.

## Authors' contributions

DF designed research, performed research, analyzed data and wrote the manuscript, AK performed research, analyzed data and prepared the manuscript. KB, SD, RT, VZ and BLS carried out research and analyzed data, PPN contributed analytical tools, JP contributed analytical tools, AB provided *RAGE^-/- ^*and *Icam1^-/- ^*mice and edited the manuscript, MS designed research and wrote the manuscript. All authors read and approved the final manuscript.
